# Transfer learning for segmentation with hybrid classification to Detect Melanoma Skin Cancer^☆^

**DOI:** 10.1016/j.heliyon.2023.e15416

**Published:** 2023-04-14

**Authors:** Ravi Dandu, M Vinayaka Murthy, Y.B. Ravi Kumar

**Affiliations:** REVA University, Bengaluru, India

**Keywords:** Colour layout filter, Auto color correlogram filter, Attribute selection classifier, Binary pattern pyramid filter, Bagging

## Abstract

Melanoma is an abnormal proliferation of skin cells that arises and develops in most of the cases on surface of skin that is exposed to copious amounts of sunlight. This common type of cancer may develop in areas of the skin that are not exposed to a much abundant sunlight. The research addresses the problem of Segmentation and Classification of Melanoma Skin Cancer. Melanoma is the fifth most common skin cancer lesion. Bio-medical Imaging and Analysis has become more promising, interesting, and beneficial in recent years to address the eventual problems of Melanoma Skin Cancerous Tissues that may develop on Skin Surfaces. The evolved research finds that Attributes Selected for Classification with Color Layout Filter model. The research has produced an optimal result in terms of certain performance metrics accuracy, precision, recall, PRC (what is PRC? Expansion is needed in Abstract), The proposed method has yielded 90.96% of accuracy and 91% percent of precise and 0.91 of recall out of 1.0, 0.95 of ROC AUC, 0.87 of Kappa Statistic, 0.91 of F-Measure. It has been noticed a lowest error with reference to proposed method on certain dataset. Finally, this research recommends that the Attribute Selected Classifier by implementing one of the image enhancement techniques like Color Layout Filter is showing an efficient outcome.

## 1 Introduction

The generic form of most cancers, on the other hand, can grow in elements of the skin that are not exposed to a lot of daylight. Melanoma is the fifth most frequent skin cancer lesion found across globe. It is considered the most dangerous kind of skin cancer according to the Skin Cancer Foundation (SCF), as it may spread to other portions of human body [1,2].

Melanoma is tough to cure if it spreads to other parts of the human body. Early detection, on the other hand, saves lives that are rare but extremely perilous. The frequency of skin cancer has been rising in recent years and statistics indicate that melanoma recurrence occurs at regular intervals. When skin cells lose control and skin disease develops, which is the most dangerous type of cancer [3]. Basal cell skin tumors also called Basal Cell Carcinomas (BCC), Squamous Cell Skin Malignancies also called Squamous Cell Carcinomas (SCC), and melanomas are of three types among skin cancer or skin tumors [4]. Skin tumor's that aren't melanomas are usually classified together as non-melanoma skin tumors. Skin cancer is classified into different classes of Melanoma Skin Cancer, which includes Malignant and Benign.

## 2 Related works

One of the AI algorithms defines training data inputs to training data outputs by constructing a complicated structure of layers for handling detailed challenges in the global health care system [[1], [2], [3]]. Deep learning algorithms are currently being used to address issues that are difficult to solve with regular ANNs [[4], [5], [6], [7]]. ANNs have been used to analyze vast quantities of scientific data, learn from complicated information [[8], [9], [10]], evolve recognition systems utilizing image processing with analysis and classification of texts [[11], [12], [13], [14], [15]] among other things.

Biomedical diagnostics is one of the most important applications of ANNs [[16], [17], [18], [19], [20]]. Furthermore, employing biological data and magnetic resonance imaging (MRI) as the diagnostic method, ANNs were employed to assess health informatics [[21], [22], [23], [24], [25]]. Artificial intelligence algorithms were used to perform a variety of biomedical tasks, including determining the specific disease areas in a biomedical segmentation image, developing diagnostic systems [[26], [27], [28]], classifying all types of diseases, predicting diseases using health informatics, and detecting numerous anatomical regions of interest [[29], [30], [31], [32]]. Deep learning algorithms have been effectively developed in the field of biomedical health [[33], [34], [35]]. Algorithms for detecting skin cancer to test their suggested research models, they examined a variety of dermatological datasets [[34], [35], [36], [37], [38], [39], [40], [41]], [42–46].

## 3 Dataset

The research has focused its attention on a benchmark dataset such as SIIM ISIC which consists of Melanoma Skin Cancer Images in DICOM format of standard dimension 1024 × 1024. The evolved research has given its contribution in terms of training the system. Firstly, with segmentation of Melanoma portion from an image with existing segmentation approach [42]. Furthermore, the research has extracted features from image with feature description mentioned in Table 1. These features are considered or learning and subjected to classification of images.

**Table 1:** Meta data of International Skin Imaging Collaboration (ISIC) dataset.

Sl. No	Name of the Class	Description
1	nv	Melanocytic nevi
2	mel	Melanoma
3	bkl	Benign keratosis lesions
4	bcc	Basal cell carcinoma
5	akiec	Actinic keratoses
6	vasc	Vascular
7	df	Dermatofibroma

## 4 Materials and Methods

The Materials and methods of this research is implemented by ISIC 2018. This work considers around 10,000 images. They are specified in below Table 1.

The purpose of using WEKA is to project the details of proposed method and to display the results of our approach on a small dataset. Furthermore, the research has gained its attention in terms of learning and testing the benchmark dataset with better performance optimization. The main objective of using these WEKA Software is display the features calculations and the result of feature measurements in terms of feature extraction and features classification. Finally, the result of classification and performance has been displayed in the form of graphical representation on WEKA.

The purpose of segmentation is to extract the features from an image and to subject it to the appropriate solution of a problem stated. The measures adopted to overcome the drawbacks of the existing approach and how effectively, the research has gained its focus on performance measures in terms of accuracy and precision vs. recall along with sensitivity and specificity.

### 4.1 Methods

The following techniques have been applied in this evolved method.

1) Apply Auto Color Correlogram Filter, Binary Patterns Pyramid Filter, and Colour Layout Filter
2) Correlate Machine Learning Algorithms
  a) Attribute selection for Classifier-Dimensionality while training and testing data reduction before being passed on to a classifier.
  b) Bagging-of-Class vis-a-vis for bagging a classifier to reduce variance.
3) In order to obtain better solution

**Fig. 1:**
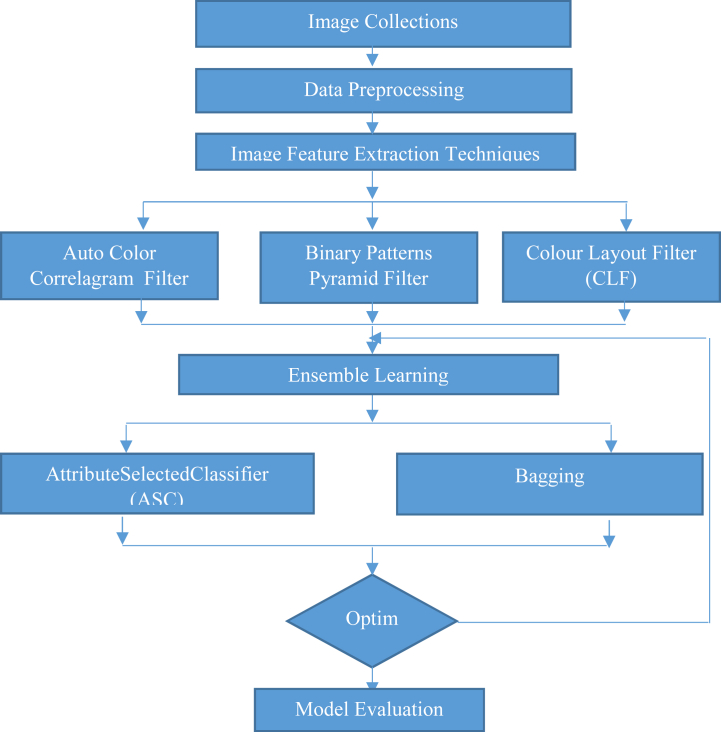
Proposed method specifies ensemble with Transfer Learning.

**Fig. 2:**
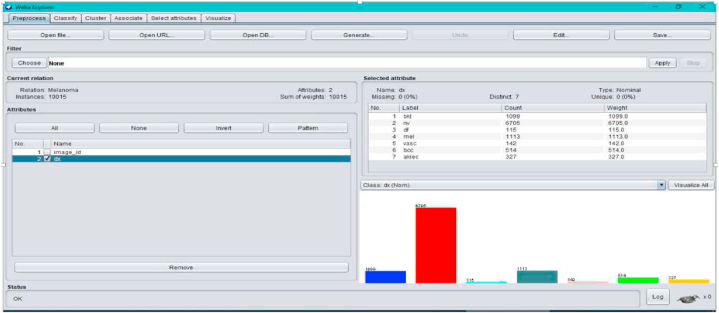
Overview of multiclass in Weka3.9.5.

**Fig. 3:**
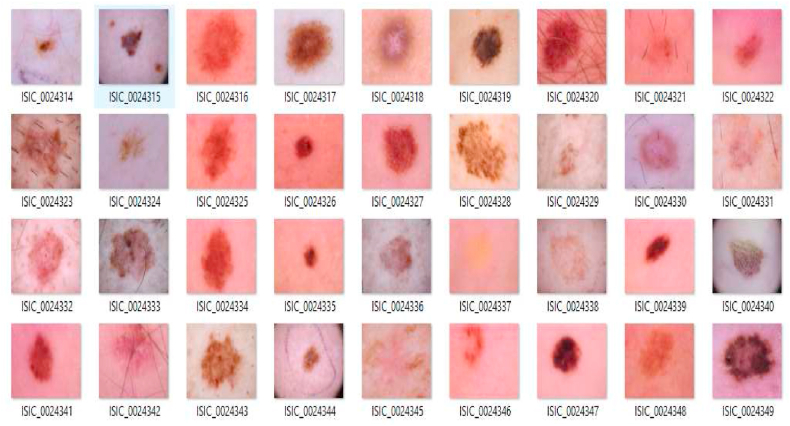
Sample Dataset used for addressing the Melanoma Skin Cancer Tissue Classification.

In order to yield better results as shown in Fig. 3, following techniques have been incorporated in open supply programme's like Weka3.9.5 (see Figs. 1 and 2). This observatory problems collected makes use of 10% of the complete dataset and makes use of ten-fold validation for all categories.

The evolved research represented in Fig. 1 has given big boosting performance to the study of Medical Image Classification, as it addresses the limitations of classifying the Melanoma Skin Cancer Images with Hybrid Classification.

The evolved research represented in Fig. 2 provides information of Medical Image Classification, as it addresses the limitations of classifying the Melanoma Skin Cancer tissues addressed with Hybrid Classification. The result of processing with Weka is shown in Fig. 2.

The sample images of dataset considered is shown in Fig. 3. The study of Medical Image Classification to address the limitations of classifying the Melanoma Skin Cancer Images with Hybrid Classification.

## 5 Results and discussions

The proposed hybrid classifier has yielded better results and given good avenues for analysis of research work tabulated in Table 2. The experimental analysis is recognized as a good Classifier in the class of Ensemble classifiers namely Bagging and Attribute Selection Classifier by using Auto Color Correlogram Filter, Binary Patterns Pyramid Filter, and Colour Layout Filter for bringing the optimal solution to the problem stated in Section II.

**Table 2:** Various measurement of classifiers on dataset by auto color correlogram filter.

Sl. No	Ensemble Classifier	Accuracy	Precision	Recall	ROC	PRC
**1**	Bagging with Auto Color Correlogram Filter	83.88%	0.85	0.84	0.92	0.90
**2**	Attribute Selected Classifier with Auto Color Correlogram Filter	82.65%	0.82	0.83	0.92	0.72
**3**	Bagging with Binary Patterns Pyramid Filter	85.06%	0.84	0.85	0.90	0.88
**4**	Attribute Selected Classifier with Binary Patterns Pyramid Filter	89.97%	0.90	0.90	0.91	0.91
**5**	Bagging with Colour Layout Filter	85.77%	0.85	0.86	0.87	0.88
**6**	Attribute Selected Classifier with Colour Layout Filter	90.96%	0.91	0.91	0.95	0.87

**Fig. 4:**
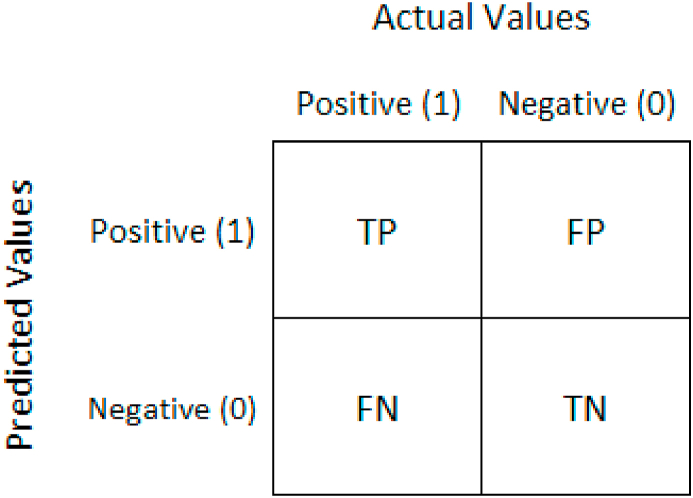
The presentation of Confusion Matrix is for assessing the performance of proposed method with existing methods.

The accuracies obtain from the selected classifiers with selected image enhancement techniques is shown in Fig. 4. The Bagging of meta category classification algorithm by using Auto Color Correlogram Filter has 83.88% of accuracy level, the Attribute Selected Classification algorithm by implementing Auto-Color Correlogram has 82.65% of accuracy, the Bagging by utilizing Binary Patterns Pyramid Filter of image feature extraction technique has produced 85.06% of accuracy, the Attribute Selected Classification algorithm by implementing Binary Patterns Pyramid Filter of image feature extraction technique has 89.97% of accuracy, the Bagging classifier using Colour Layout Filter of Image enhancement technique has 85.77% of accuracy and the penultimate Attribute Selected Classification algorithm using Colour Layout Filter of Image enhancement technique has 82.65% of accuracy.

**Fig. 5:**
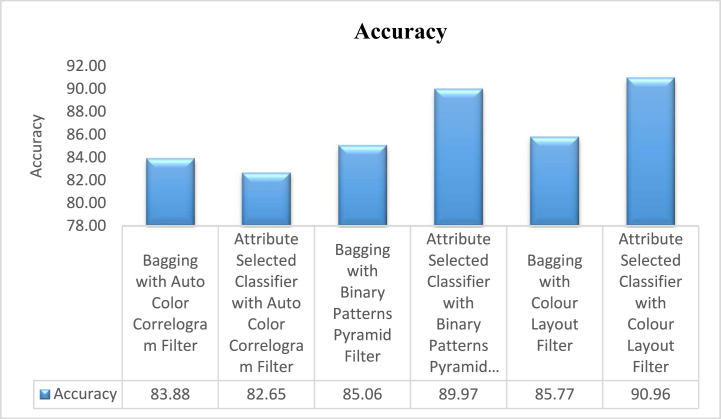
Graphical representations of various classifiers with their accuracy levels.

The precision values obtain from the selected classifiers with selected image enhancement techniques is shown in Fig. 5. The Bagging of meta category classification algorithm by using Auto Color Correlogram Filter has 0.85 of precision, the Attribute Selected Classification algorithm by implementing Auto Color Correlogram has 0.82 precision, the Bagging by utilizing Binary Patterns Pyramid Filter of image feature extraction technique has 0.84 of precision, the Attribute Selected Classification algorithm by implementing Binary Patterns Pyramid Filter of image feature extraction technique has 0.90 of precision level, the Bagging classifier using Colour Layout Filter of Image enhancement technique has 0.85 precision level, and the penultimate Attribute Selected Classification algorithm using Colour Layout Filter of Image enhancement technique has 0.91 precision.

**Fig. 6:**
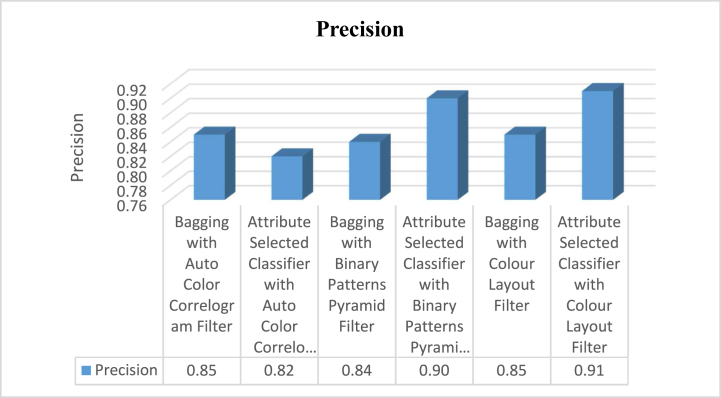
Graphical representations of various classifiers with their precision values.

The memory values acquire from the particular classifiers with selected image enhancement techniques is shown in Fig. 6. The Bagging of meta category classification algorithm by using Auto-Color Correlogram Filter has 0.84 of recall, the Attribute Selected Classification algorithm by implementing Auto-Color Correlogram has 0.83 recall, the Bagging by utilizing Binary Patterns Pyramid Filter of image feature extraction technique has 0.85 recall, the Attribute Selected Classification algorithm by implementing Binary Patterns Pyramid Filter of image feature extraction technique has 0.90 recall, the Bagging classifier using Colour Layout Filter of Image enhancement technique has 0.86 recall, and the Attribute Selected Classification algorithm using Colour Layout Filter of Image enhancement technique has 0.91 recall.

**Fig. 7:**
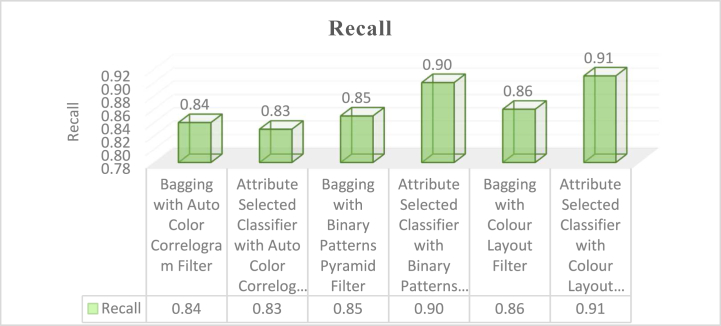
Graphical representations of various classifiers with their recall values.

The ROC values obtain from selected classifiers with selected image enhancement techniques is shown Fig. 7. The Bagging of meta category classification algorithm by using Auto Color Correlogram Filter has 0.92 of ROC level, the Attribute Selected Classification algorithm by implementing Auto Color Correlogram has 0.92 of ROC level, the Bagging by utilizing Binary Patterns Pyramid Filter of image feature extraction technique has 0.90 of ROC level, the Attribute Selected Classification algorithm by implementing Binary Patterns Pyramid Filter of image feature extraction technique has 0.91 of ROC level, the Bagging classifier using Colour Layout Filter of Image enhancement technique has 0.87 of ROC level, and the Attribute Selected Classification algorithm using Colour Layout Filter of Image enhancement technique has 0.95 of ROC level.

The precisions obtain from the nominated classifiers with selected image feature extraction techniques has shown in Fig. 4. This graph signifies that the contrasts of precisions for all the groups of the classifiers with various image filters. The least accuracy value is 82.65% which is produced by Attribute Selected Classifier algorithm by using Auto Color Correlogram Filter. The Highest accuracy value is 90.96% which is having Attribute Selected Classifier by implementing Colour Layout Filter. The Bagging with Auto Color Correlogram Filter, Bagging using Binary Patters Pyramid Filter, bagging algorithm using Colour Layout Filter and Attribute Selected Classifier algorithm using Binary Patters Pyramid Filter are having accuracy values from 83.88% of accurateness level to 89.97% of accurateness level.

The precision values obtain from the selected classifiers with selected image feature extraction techniques has exposed in Fig. 5. This graph characterizes that the comparisons of precision values for all the groups of the classifiers with various image filters. The least precision value is 0.82 which is produced by Attribute Selected Classifier algorithm by using Auto Color Correlogram Filter. The Highest precision value is 0.91 which is having Attribute Selected Classifier by implementing Colour Layout Filter. The rest of the classifiers like, the Bagging with Auto Color Correlogram Filter, Bagging using Binary Patters Pyramid Filter, Bagging algorithm using Colour Layout Filter and Attribute Selected Classifier algorithm using Binary Patters Pyramid Filter are having precision values from 0.85 of precision level to0.90 of precision level.

**Fig. 8:**
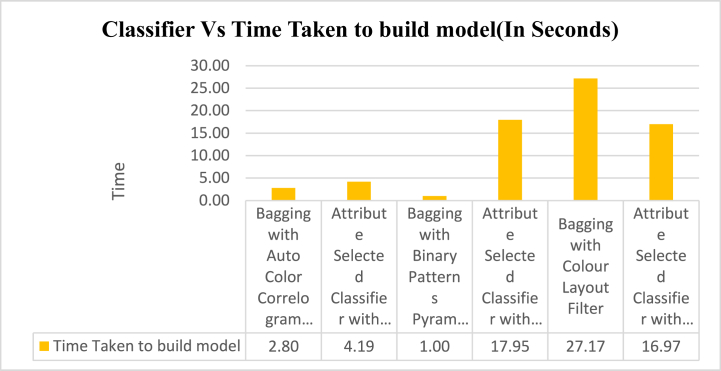
Graphical representations of various Classifiers with their time is taken to build a model.

The memory values acquire from the particular classifiers with selected image feature extraction techniques has exposed in Fig. 6. This graph signifies that the contrasts of recall values for all the groups of the classifiers with various image filters. The least recall value is 0.83 which is produced by Attribute Selected Classifier algorithm by using Auto Color Correlogram Filter. The Highest recall value is 0.91 which is having Attribute Selected Classifier by implementing Colour Layout Filter. The rest of the classifiers like, the Bagging with Auto Color Correlogram Filter, Bagging using Binary Patters Pyramid Filter, Bagging algorithm using Colour Layout Filter and Attribute Selected Classifier algorithm using Binary Patters Pyramid Filter are having recall values from 0.84 of recall level to 0.90 of recall level. The strengths of our proposed method shall be noticed in terms of performance measures. On the other hand, the weaknesses of our approach may be time complexity compared with contemporary approaches. Fig. 8 indicates the results of time taken for processing an image of a dataset.

## 6 Conclusion

**Fig. 9:**
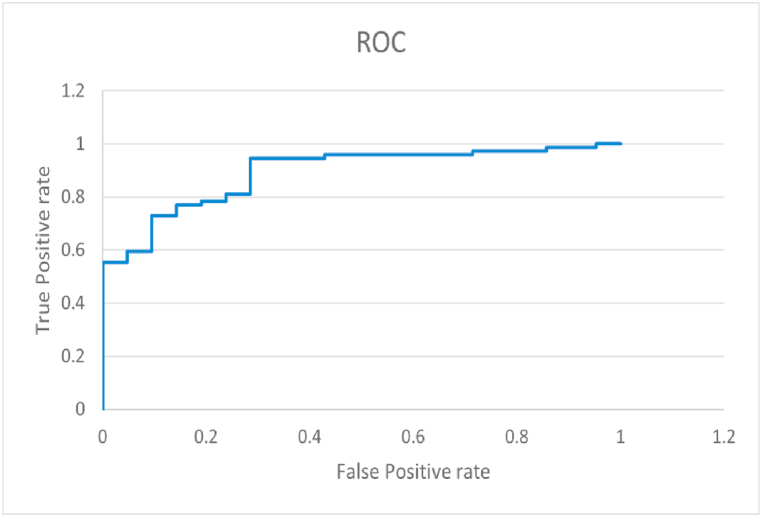
ROC of SVM Classification included for assessing the dataset SIIM ISIC 2018.

This research work finds that the Attribute Selected Classifier of ensemble category using Color Layout Filter model is producing the efficient output with less error values such as accuracy, precision, recall, ROC Fig. 9 with AUC has yielded 90.96%, 0.91, 0.95, 0.87 respectively. The evolved results have shown significant contribution to classification of Melanoma Skin Images into different stages of Skin Cancer, which is having Attribute Selected Classifier by implementing Colour Layout Filter. This research work recommends that the Attribute Selected Classifier of ensemble category using Color Layout Filter.

## Role of funding source

No funding source.

## Author contribution statement

1 – Ravi Dandu: Conceived and designed the experiments; Wrote the paper; 2 – Vinayaka Murthy M: Performed the experiments; 3 – Ravi Kumar Y B: Analyzed and interpreted the data, Contributed analysis tools or data.

## Data availability statement

Data associated with this study has been deposited at XXX under the accession number YYY. Where, XXX refers to the link https://challenge2020.isic-archive.com under the accession number YYY refers to the id associated with this DOI: https://doi.org/10.34970/2020-ds01.

## Declaration of competing interest

The Author(s) declares(s) that there is no conflict of interest.

## References

[1] Alsaade Fawaz Waselallah (2021). Developing a recognition system for diagnosing melanoma skin lesions using artificial intelligence algorithms. Hindawi Comput. Math. Methods Med..

[2] https://dataverse.harvard.edu/dataset.xhtml?persistentId=doi:10.7910/DVN/DBW86T.

[3] (2017). Key statistics for melanoma skin cancer. https://www.cancer.org/cancer/melanoma-skin-cancer/about/key-statistics.html.

[4] Jain S., Pise N. (2015). Computer aided melanoma skin cancer detection using Image Processing. Proc. Comput. Sci..

[5] Masood A., Ali Al-Jumaily A. (2013). Computer aided diagnostic support system for skin cancer: a review of techniques and algorithms. Int. J. Biomed. Imag..

[6] Aldhyani T.H.H., Alrasheed M., Al-Adaileh M.H., Alqarni A.A., Alzahrani M.Y., Alahmadi A.H. (2021). Deep learning and holt-trend algorithms for predicting covid-19 pandemic. Comput. Mater. Continua (CMC).

[7] Herman C. (2012). Emerging technologies for the detection of melanoma: achieving better outcomes. Clin. Cosmet. Invest. Dermatol..

[8] Alsaade F.W., Aldhyani T.H.H., Al-Adhaileh M.H. (2021). Developing a recognition system for classifying covid-19 using a convolutional neural network algorithm. Comput. Mater. Continua (CMC).

[9] LeCun Y., Bengio Y., Hinton G. (2015). Deep learning. Nature.

[10] Aleid M.A., Alyamani K.A.Z., Rahmouni M., Aldhyani T.H.H., Alsharif N., Alzahrani M.Y. (2021). Modelling the psychological impact of covid-19 in Saudi Arabia using machine learning. Comput. Mater. Continua (CMC).

[11] Senan E.M., Alsaade F.W., Al-Mashhadani M.I.A., Aldhyani T.H.H., Al-Adhaileh M.H. (2020). Classification of histopathological images for early detection of breast cancer using deep learning. J. Appl. Sci. Eng..

[12] Shin H.C., Roth H.R., Gao M. (2016). Deep convolutional neural networks for computer-aided detection: CNN architectures, dataset characteristics and transfer learning. IEEE Trans. Med. Imag..

[13] Song J., Qin S., Zhang P. (2016). 2016 IEEE/ACIS 15th International Conference on Computer and Information Science (ICIS).

[14] Lee J.G., Jun S., Cho Y.W. (2017). Deep learning in medical imaging: general overview. Korean J. Radiol..

[15] Suzuki K. (2017). Overview of deep learning in medical imaging. Radiol. Phys. Technol..

[16] Ravì D., Wong C., Deligianni F. (2017). Deep learning for health informatics. IEEE J. Biomed. HealthInf..

[17] Mamoshina P., Vieira A., Putin E., Zhavoronkov A. (2016). Applications of deep learning in biomedicine. Mol. Pharm..

[18] Liu J., Pan Y., Li M. (2018). Applications of deep learning to MRI images: a survey. Big Data Min. Anal..

[19] Zhou H., Schaefer G., Sadka A., Celebi M.E. (2009). Anisotropic mean shift based fuzzy c-means segmentation of dermoscopy images. IEEE J. Selected Top. Signal Process..

[20] Zhou H., Schaefer G., Celebi M.E., Lin F., Liu T. (2011). Gradient vector flow with mean shift for skin lesion segmentation. Comput. Med. Imag. Graph..

[21] Zhou H., Li X., Schaefer G., Celebi M.E., Miller P. (2013). Mean shift based gradient vector flow for image segmentation. Comput. Vis. Image Understand..

[22] Garnavi R., Aldeen M., Celebi M.E., Varigos G., Finch S. (2011). Border detection in dermoscopy images using hybrid thresholding on optimized color channels. Comput. Med. Imag. Graph..

[23] Pennisi A., Bloisi D.D., Nardi D., Giampetruzzi A.R., Mondino C., Facchiano A. (2016). Skin lesion image segmentation using delaunay triangulation for melanoma detection. Comput. Med. Imag. Graph..

[24] Ma Z., Tavares J. (2017). A novel approach to segment skin lesions in dermoscopic images based on a deformable model. IEEE J. Biomed. Health Inf..

[25] Yu L., Chen H., Dou Q., Qin J., Heng P.A. (2017). Automated melanoma recognition in dermoscopy images via very deep residual networks. IEEE Trans. Med. Imag..

[26] Celebi M.E., Kingravi H.A., Uddin B. (2007). A methodological approach to the classification of dermoscopy images. Comput. Med. Imag. Graph..

[27] Celebi M.E., Iyatomi H., Schaefer G., Stoecker W.V. (2009). Lesion border detection in dermoscopy images. Comput. Med. Imag. Graph..

[28] Schaefer G., Krawczyk B., Celebi M.E., Iyatomi H. (2014). An ensemble classification approach for melanoma diagnosis. Memetic Comput..

[29] Wolner Z.J., Yélamos O., Liopyris K., Rogers T., Marchetti M.A., Marghoob A.A. (2017). Enhancing skin cancer diagnosis with dermoscopy. Dermatol. Clin..

[30] Wichakam I., Vateekul P. (2016). Proceedings of the 8th International Conference on Knowledge and Smart Technology.

[31] Chan T.F., Vese L.A. (2001). Active contours without edges. IEEE Trans. Image Process..

[32] Zhang K., Zhang L., Song H., Zhou W. (2010). Active contours with selective local or global segmentation: a new formulation and level set method. Image Vis. Comput..

[33] M. George and R. Zwiggelaar, “Breast tissue classification using Local Binary Pattern variants: a comparative study,” in Medical Image Understanding and Analysis. MIUA 2018. Communications in Computer and Information Science, vol. 894 pp. 143–152, Springer, Cham.

[34] Lundervold A.S., Lundervold A. (2019). An overview of deep learning in medical imaging focusing on MRI. Z. Med. Phys..

[35] Shrestha A., Mahmood A. (2019). Review of deep learning algorithms and architectures. IEEE Access.

[36] Ravi Kumar Y.B., Ravi Kumar C.N. (2016). Harmonic rule for measuring the facial similarities among relatives. Trans. Mach. Learn. Artif. Intell..

[37] Ravi Kumar Y.B., Narayanappa C.K. (2018). Triangular similarities of facial features to determine: the relationship among family members. J. Adv. Comput. Intell. Intell. Inf..

[38] Ravi Kumar Y.B., Narayanappa C.K., Dayananda P. (2020).

[39] Hosny K.M., Kassem M.A. (2022). Refined residual deep convolutional network for skin lesion classification. J. Digit. Imag..

[40] Hosny, K.M., Kassem, M.A. & Foaud, M.M. Skin melanoma classification using ROI and data augmentation with deep convolutional neural networks. Multimed. Tool. Appl. 79, 24029-24055.

[41] Kassem M.A., M Hosny K., Damaševičius R., Eltoukh Mohamed M. (2021). Machine learning and deep learning methods for skin lesion classification and diagnosis: a systematic review. Diagnostics.

